# It Takes a Team to Make It Through: The Role of Social Support for Survival and Self-Care After Allogeneic Hematopoietic Stem Cell Transplant

**DOI:** 10.3389/fpsyg.2021.624906

**Published:** 2021-03-26

**Authors:** Yaena Song, Stephanie Chen, Julia Roseman, Eileen Scigliano, William H. Redd, Gertraud Stadler

**Affiliations:** ^1^Department of Health and Behavior Studies, Teachers College, Columbia University, New York, NY, United States; ^2^Department of Psychology, Columbia University, New York, NY, United States; ^3^Health and Human Sciences, Charité – Universitätsmedizin Berlin, Berlin, Germany; ^4^Mount Sinai Hospital, New York, NY, United States; ^5^Icahn School of Medicine at Mount Sinai, New York, NY, United States; ^6^Applied Health Sciences, University of Aberdeen, Aberdeen, United Kingdom

**Keywords:** allogeneic hematopoietic cell transplant, cancer, caregiver support, healthcare providers, multiple medication adherence, social support, survival, self-care

## Abstract

**Background:**

Social support plays an important role for health outcomes. Support for those living with chronic conditions may be particularly important for their health, and even for their survival. The role of support for the survival of cancer patients after receiving an allogeneic hematopoietic cell transplant (alloHCT) is understudied. To better understand the link between survival and support, as well as different sources and functions of support, we conducted two studies in alloHCT patients. First, we examined whether social support is related to survival (Study 1). Second, we examined who provides which support and which specific support-related functions and tasks are fulfilled by lay caregivers and healthcare professionals (Study 2).

**Methods:**

In Study 1, we conducted a retrospective chart review of alloHCT patients (*N* = 173, 42.8% female, age: *M* = 49.88) and registered availability of a dedicated lay caregiver and survival. In Study 2, we prospectively followed patients after alloHCT (*N* = 28, 46.4% female, age: *M* = 53.97, 46.4% ethnic minority) from the same hospital, partly overlapping from Study 1, who shared their experiences of support from lay caregivers and healthcare providers in semi-structured in-depth interviews 3 to 6 months after their first hospital discharge.

**Results:**

Patients with a dedicated caregiver had a higher probability of surviving to 100 days (86.7%) than patients without a caregiver (69.6%), OR = 2.84, *p* = 0.042. Study 2 demonstrated the importance of post-transplant support due to patients’ emotional needs and complex self-care regimen. The role of lay caregivers extended to many areas of patients’ daily lives, including support for attending doctor’s appointments, managing medications and financial tasks, physical distancing, and maintaining strict dietary requirements. Healthcare providers mainly fulfilled medical needs and provided informational support, while lay caregivers were the main source of emotional and practical support.

**Conclusion:**

The findings highlight the importance of studying support from lay caregivers as well as healthcare providers, to better understand how they work together to support patients’ adherence to recommended self-care and survival.

## Introduction

Social support has long been recognized as a key contributor to health (Berkman et al., 2000; Uchino, 2006; Holt-Lunstad et al., 2010). It is associated with higher quality of life and even survival in cancer (Chou et al., 2012; Aizer et al., 2013; Luszczynska et al., 2013). For recipients of an allogeneic hematopoietic cell transplant (alloHCT, commonly used to treat blood and lymphoid cancers), social support is especially critical. Many hematopoietic cell transplant programs require a caregiver to become eligible for transplant (National Marrow Donor Program, 2017; Preussler et al., 2019). However, so far, there is conflicting evidence regarding the impact of social support on survival after alloHCT.

A systematic review by Beattie et al. (2013) contained six studies published before 2011 in this population, while we identified six additional recent studies in our literature update. Four studies found that patients with social support after transplant had higher rates of survival than those without support (Colón et al., 1991: *N* = 100 patients after alloHCT, 55 vs. 20% at 24 months; Frick et al., 2005: *N* = 99 patients after autologous transplant, 78 vs. 40% at 47 months; Foster et al., 2005: *N* = 131 patients after alloHCT, 54 vs. 15%; Foster et al., 2013, also reported in McLellan et al., 2011: *N* = 164 alloHCT patients, 42 vs. 26%). Foster et al., 2013 highlighted the importance of longer and more frequent visits from a dedicated lay caregiver for survival, contrasted with merely having a support system. Another study (*N* = 92, 46% after alloHCT and 54% after autologous transplant) did not report enough information to calculate survival rates but found higher survival with better support (Rodrigue et al., 1999). AlloHCT patients with at least one close and dependable relationship partner survived for longer after transplant than those with poorer support pre-transplant (*N* = 400, *HR* = 0.57 over 2 years; Ehrlich et al., 2016). Patients after alloHCT who were single showed shorter survival times than those married or in committed relationships (*N* = 130 over a median follow-up of 713 days, *HR* = 1.91; Pillay et al., 2014). Another recent study found a non-significant tendency that support stability and support availability were related to survival in a smaller sample of 119 patients after alloHCT (*HR* = 1.29 and 1.23 over a median follow-up of 721 days; Harashima et al., 2019).

While considerable evidence indicates that support matters, some studies found no link between support or marital status and survival. An unpublished dissertation with a large sample did not find an association between support and survival (*N* = 272 patients, 83% after alloHCT, 17% after autologous transplant; Artherholt, 2007). Three additional studies that used marital status as a support indicator failed to find a link with survival in large samples of patients after alloHCT (*N* = 10,226, Tay et al., 2020; *N* = 715, Gerull et al., 2017; *N* = 309; Sato et al., 2018). However, two of these studies still found some evidence that social support could matter for survival: Tay et al. (2020) found an association for graft-versus-host disease (GvHD), while Gerull et al. (2017) found that patients with missing information on marital status had worse survival than those with available information.

The available evidence leaves considerable gaps. Larger studies relied on marital status as a support indicator, while smaller studies used more elaborate and nuanced measures. Overall, social support was not consistently measured, with marital status likely being too coarse a measure for support, as it ignores other sources than the spouse (such as parents, siblings, or children, Foster et al., 2013; Sato et al., 2018; Preussler et al., 2019). Taken together, the mixed outcomes of the available studies and varying indicators of social support suggest the need for a deeper understanding of the characteristics of caregivers and functions of social support in patients after alloHCT (Beattie et al., 2013; Tay et al., 2019).

Patients after transplant experience high mortality due to potentially life-threatening complications, infections, GvHD, and cancer recurrence (Pasquini and Wang, 2011; Holtan et al., 2015). Patients are prescribed a complex self-care regimen to improve survival rates, including procuring and taking 18 or more different medications, frequent hospital visits, and following strict dietary, hydration, and hygiene requirements including social distancing (Tomblyn et al., 2009; Morrison et al., 2017). However, adherence to this complex regimen has not been ideal. A study following 376 alloHCT recipients found that almost two-thirds were non-adherent in taking immunosuppressant medication (Kirsch et al., 2014), while 54.6% of alloHCT patients were poorly adherent to their medication regimens in a recent pilot study (Lehrer et al., 2018).

Reviews of the existing literature, examining both structural and functional support, found that social support, especially practical support provided by close others, was linked to better adherence (DiMatteo, 2004; Scheurer et al., 2012). A recent review of 52 studies in hematological cancer patients found that social support was associated with medication adherence (Hall et al., 2016). A study of 21 alloHCT patients and their partners found varying rates (19 to 100%) of adherence to various post-transplant self-care tasks, with adherence levels dependent on which dyad member was responsible for the task (Posluszny et al., 2018). There are few studies examining support from healthcare providers (Hall et al., 2016), with available studies suggesting that patient-physician communication and relationship quality are linked to patient adherence to treatment and medications (Haskard Zolnierek and DiMatteo, 2009; Hillen et al., 2010; Espinosa and Kadić-Maglajlić, 2019).

We conducted two studies in alloHCT patients to address gaps in the literature. In Study 1, we conducted a retrospective chart review to examine whether social support is related to survival. In Study 2, we conducted a prospective study at the same hospital to examine characteristics of lay caregivers and healthcare providers and the types of support they provide to alleviate distress and facilitate patients’ adherence to prescribed self-care.

## Study 1: Support and Survival in AlloHCT Patients

In Study 1, we conducted a retrospective chart review of alloHCT patients.

### Methods

#### Setting and Participants

The study took place at a large urban teaching hospital which serves a diverse population and has a well-established transplant program delivering alloHCT. The program requires the patient to name a caregiver to become eligible for alloHCT. The research team conducted a retrospective chart review study. Patients were eligible for inclusion in the chart review if they were at least 18 years old and had received their first alloHCT between February 26, 2009 and August 28, 2013. The research team then coded survival data for up to 6 months until February 28, 2014. Patients with identical dates for birth, treatment receipt, or death were individually examined and duplicates due to data entry errors removed, resulting in data from 173 participants eligible for inclusion in these analyses. The local Institutional Review Board of the hospital from which the data were collected reviewed and approved the study (HS# 13-00761).

#### Measures

Members of the hospital team abstracted information from patient charts, including patient background information on age, gender, minority background, cancer type, and transplant donor type. *Support* was assessed with data from the hospital database (Epic). A member of the research team consulted the “next of kin” information on the first page of each patient chart (“snapshot view”) that the study team used in Study 2 to contact caregivers to schedule appointments; it is also used in clinical care, for example, to inform others should the patient pass away or in case of financial matters. This field is filled out by the admission team when a patient is admitted for transplant. We coded support available if there was someone listed in this field (0 = no, 1 = yes). Additionally, if there was no one listed or if the person listed didn’t match up with the clinicians’ recollection of the caregiver, the staff member read through the Social Work notes, and coded “1” if there was mention of a caregiver consistently involved or 0 if there was no one listed. Due to the placement on the first page of each patient chart, its use for clinical work as well as hospital finances, and our own observation of its validity in Study 2, we assume that the coded information reflects the availability of support to patients after alloHCT.

*Survival* was calculated based on the number of days between first transplant and the date when the chart review was completed (February 28, 2014), if the patient was still alive, or date of death, if the patient had died before that point in time. Survival to 100 days and to 180 days was coded as a binary variable (0 = no, 1 = yes). Because patients receive continuing care at the transplant clinic over an extended period of time and the clinic reported patient outcomes to a national database, the abstracting team was able to resolve nearly all issues with missing data or data entry errors.

#### Data Analysis

We ran logistic regressions predicting survival to 100 days and to 180 days, with the main predictor availability of a caregiver (caregiver available: 1 = yes, 0 = no). In additional analyses, we adjusted for covariates which are relevant for survival, such as age (centered at the grand mean), gender (also centered at the grand mean), minority background (1 = yes, 0 = no), cancer type (leukemia 1 = yes, 0 = no), and transplant donor (with HLA-identical sibling transplant, coded 1 = yes, 0 = no). All analyses were conducted with IBM SPSS version 26.0 with a significance level of *p* < 0.05.

### Results

#### Characteristics of Patients

In total, 173 patients (42.8% female, age: *M* = 49.88) received an alloHCT for the first time in the chart review period. The sample was ethnically diverse, with half of the participants coming from a minority background (*n* = 90, 52.0%; Non-Hispanic White: 48.0%, African American: 10.4%, Asian: 15.6%, Hispanic: 26.0%. A majority of patients had leukemia as cancer type (85.0%). The allogeneic cells for the transplant came in one of three patients from an HLA-identical sibling (36.4%).

#### Support and Survival

Patients’ hospital files mentioned a dedicated caregiver in the patient chart for 150 patients (88.2%), while 23 patients (11.2%) did not have a dedicated caregiver listed. Of the 173 patients analyzed, 146 (84.4%) survived to 100 days after their first transplant, and 123 (71.1%) to 180 days.

Patients with a dedicated caregiver had a higher probability of surviving to 100 days (86.7%) than patients without a caregiver (69.6%), as logistic regression analysis showed, *OR* = 2.84, *p* = 0.042. Multiple logistic regression indicated that this effect was robust, OR = 3.03, *p* = 0.044, adjusting for covariates that explain variation in survival, such as age, gender, minority ethnic background, cancer type, and HLA-identical sibling transplant (see Tables 1 and 2 Part a). In line with prior studies, younger patients and those who were able to obtain an HLA-identical sibling transplant showed a higher probability of surviving to 100 days.

**TABLE 1:** Availability of caregiver and frequencies and probabilities of surviving to 100 and 180 days after transplant.

		Survival		Total	Survival probability (%)
		No	Yes		
**(a) Surviving to 100 days**					
Caregiver	No	7	16	23	69.6
	Yes	20	130	150	86.7
	Total		146	173	
**(b) Surviving to 180 days**					
Caregiver	No	10	13	23	56.5
	Yes	40	110	150	73.3
	Total		123	173	

**TABLE 2:** Availability of caregiver and surviving to 100 and 180 days after transplant, logistic regression without and with adjusting for covariates (*N* = 173).

	*b*	SE	Wald	df	*p*	OR	95% CI	
							Lower	Upper
(a) Surviving to 100 days								
Univariate logistic regression								
Intercept	0.83	0.45	3.33	1	0.068	2.29		
Caregiver	1.05*	0.51	4.15	1	0.042	2.84	1.04	7.77
Multiple logistic regression								
Intercept	1.56	0.93	2.80	1	0.094	4.76		
Caregiver	1.11*	0.55	4.05	1	0.044	3.03	1.03	8.90
Age	–0.43	0.19	4.88	1	0.027	0.65	0.45	0.95
Gender	–0.41	0.46	0.79	1	0.375	0.67	0.27	1.63
Ethnic minority	–0.53	0.50	1.13	1	0.289	0.59	0.22	1.56
Cancer type	–0.71	0.82	0.74	1	0.388	0.49	0.10	2.46
HLA-identical sibling transplant	1.49	0.59	6.46	1	0.011	4.44	1.41	14.01
(b) Surviving to 180 days								
Univariate logistic regression								
Intercept	0.26	0.42	0.39	1	0.533	1.30		
Caregiver	0.75	0.46	2.66	1	0.103	2.12	0.86	5.21
Multiple logistic regression								
Intercept	0.44	0.67	0.43	1	0.513	1.55		
Caregiver	0.77	0.47	2.66	1	0.103	2.15	0.86	5.42
Age	–0.16	0.14	1.26	1	0.261	0.85	0.65	1.13
Gender	–0.01	0.36	0.00	1	0.968	0.99	0.49	2.00
Ethnic minority	0.21	0.38	0.31	1	0.580	1.24	0.58	2.62
Cancer type	–0.54	0.55	0.99	1	0.320	0.58	0.20	1.70
HLA-identical sibling transplant	0.56	0.38	2.25	1	0.134	1.76	0.84	3.66

*b, regression weight; SE, standard error; Df, degrees of freedom; p, significance level; OR, odds ratio; CI, confidence interval with lower and upper limit.*

At 180 days, more patients had passed away, and the effect sizes were somewhat smaller and non-significant, yet showed the same pattern of results: Patients with a dedicated caregiver had higher chances of surviving to 180 days (73.3%) than patients without a caregiver (56.5%), OR = 2.12, *p* = 0.103, with nearly identical effect size after adjusting for covariates, OR = 2.15, *p* = 0.103 (see Tables 1, 2, Part b).

### Discussion

Study 1 found that the availability of a dedicated caregiver was related to a 17.1% higher rate of surviving to 100 days after transplant, compared to transplant recipients who did not have a dedicated caregiver. Support from a dedicated caregiver in the first months after leaving the hospital seems particularly important, as patients are learning to adapt to the complex self-care regimen. The effect size for survival at 180 days was similar although not significant. The findings are in line with the previous literature, demonstrating that the presence of a lay caregiver was an important factor for survival after transplant (Foster et al., 2005, 2013; McLellan et al., 2011; Beattie et al., 2013) and related to longer survival after transplant (Hoodin et al., 2006; Pillay et al., 2014). We assume that the patients who had a dedicated caregiver recovered better and survived at higher rates after transplant because they were more able to follow the complex life-saving self-care regimen with the help from their caregivers (Ehrlich et al., 2016; Posluszny et al., 2018). In order to better understand the role of support in self-care, Study 2 examined the sources and functions of social support in depth.

## Study 2: A Closer Look at Support for AlloHCT Patients’ Self-Care

Study 2 zooms in on the characteristics, support functions and tasks of lay caregivers and healthcare providers.

### Methods

#### Setting and Participants

Study 2 is a prospective qualitative study in the same patient population at the same hospital as in Study 1 with partly overlapping participants. During the study period, 84 adult patients received an alloHCT at the study site and they were all invited to participate in the study by the doctors. 7 patients (8.3%) were ineligible for participation because of language barriers (all materials were available in English, Spanish, and Mandarin). The recruitment rate was high, as about half (*n* = 38, 49.4%) of the eligible patients (*n* = 77, 91.7%) agreed to participate and signed consent. However, of the consented patients (*n* = 38), five patients did not participate in the data collection: One patient passed away prior to discharge; one patient had a complicated recovery with many hospitalizations; and three patients withdrew from the study before discharge. Data collection began with 33 patients (42.9%), but five patients were not interviewed for the following reasons: three patients deceased before interview, one was not available for interview and another one was too ill to conduct interview. Thus, we included 28 patients (84.8% of those from whom we started collecting data) in our qualitative analyses.

We interviewed patients (*N* = 28) three to 6 months after their first discharge from the hospital after receiving an alloHCT. Eligible patients were invited by healthcare providers to participate in the study during pre-transplant visits or during their hospitalization after transplant. Individuals were eligible for the study if they were blood or lymphoid cancer patients, scheduled to receive an alloHCT, at least 18 years old and spoke English, Spanish or Mandarin. As already mentioned in Study 1, the hospital requires naming a caregiver for eligibility for alloHCT. Two of the interviews were conducted in Spanish (7.1%), the rest in English (*n* = 26, 92.9%) and none in Mandarin. Participation was voluntary. All patients were prescribed a multiple medication regimen (typically consisting of 18 or more different medications with 24 or more pills per day to prevent or treat GvHD, fungal and bacterial infection, and irritation of the digestive system) as a part of the self-care regimen after discharge which also included frequent hospital visits, and abiding by strict dietary and hydration requirements and hygiene regimens including social distancing (Tomblyn et al., 2009; Morrison et al., 2017). The local Institutional Review Board of the hospital from which the data were collected reviewed and approved the study (HS# 12-00453). All participants provided written informed consent prior to participation. The study team complied with the relevant standards in reporting results (Tong et al., 2007, 2012; Creswell et al., 2011; O’Brien et al., 2014) and followed the COREQ guideline (Tong et al., 2007) closely (see Appendix A).

#### Measures

Semi-structured in-depth interviews were conducted. Interviewers followed a structured interview guide (see Appendix B) and probed individuals for detailed answers about their self-care regimen and social support. The interview guide was developed by the principal investigator, who is an expert in health psychology. Interview questions covered various domains related to individuals’ self-care regimen, including questions on social support from lay caregivers and healthcare providers. The interview guide contained the following main questions (see Appendix B for a complete list including additional probes).

• Can you share with me a little bit about how life has changed for you since your transplant?
• Who in your life knows that you are taking medication? Are there people in your life who support you taking your medications? Are there people in your life who help to make sure that you take your medication on time?
• How have your eating and drinking habits changed since the transplant?
• Does anyone help you with the pillbox? Who, specifically, helps you? How do they help you?
• Does a lack of money ever make it difficult for you to take your medication? Can you tell me about this? What do you do about it?
• Tell me about your healthcare provider(s). Who is the person who primarily treats you? What is your relationship like with your care provider? Do you think your relationship with your care provider makes it easier or harder to take your medication? How so? Do you feel like your care provider understands your needs?

To ensure the quality of the interviews, interviewers were trained by the principal investigator; the interview questions were pilot tested during role-play interviews prior to conducting actual interviews with patients. All research assistants at the time had a bachelor’s or master’s degree in social sciences, public health, or health sciences.

#### Procedures

Participants who agreed to participate in the study and signed written informed consent forms were contacted in advance to schedule each interview. Individual semi-structured interviews were conducted between three to 6 months after patients’ first discharge from hospital. Interviews were conducted either in person before hospital appointments and/or via telephone when participants could not manage hospital visits or preferred telephone interviews. In some cases, the caregiver also joined the interview when accompanying the participant to appointments. Interviews lasted from 40 to 60 min and participants could take a break if desired. All interviews were conducted by two interviewers, with one interviewer asking questions and the other taking notes and recording the interview. All the interviews were audio recorded and transcribed. The audio recordings were deleted after transcription. Data were kept in a secure password protected drive. Participant were made aware of and agreed to being recorded at the beginning of the interview. All confidential information in the interview transcripts was removed and replaced with generic titles (e.g., nurse Jane Doe with Nurse 1).

#### Data Analysis

Data on support from healthcare providers was missing for two of the 28 participants because the participants felt too sick or could not finish the interview for other reasons. Thus, percentages are calculated based on 26 interviews for information regarding support from healthcare providers. All interview transcripts were read carefully and coded in NVivo version 11. One of the authors (YS) selected seven interviews from the 28 patients (25%) to represent the sample as best as possible and two coders (YS and SC) coded individually if an interview contained information about support from lay caregivers and healthcare providers. The coders met several times with the project lead (GS) to go over the coding and discuss the discrepancies until the two were in agreement. We calculated kappa coefficient based on a binary coding if support from a lay caregiver and a healthcare provider was mentioned by the patient (mentioned: 1 = yes, 0 = no). Kappa coefficients for support from lay caregivers and support from healthcare providers were calculated separately. The kappa for sources of support (lay caregivers’ support mentioned, yes/no; healthcare provider support mentioned: yes/no) was in agreement (κ = 1.00). The finer coding of the subcategories of social support used thematic analysis (Braun and Clarke, 2006). The coding scheme initially followed classical social support theory, including instrumental, emotional and informational support (Weiss, 1974; Cohen and McKay, 1984; House et al., 1985; Thoits, 2011). Two coders (YS and SC) coded relevant quotes on social support. to identify the meanings in the interviews and analyze the data. While reading through the interviews for coding the coders identified additional themes, so added codes for the following themes: meaning in life, financial support from lay caregivers, and support with medical needs from healthcare providers. An initial category with the working title “Lack of support” was modified and renamed to ambivalence about support. A codebook was developed listing different themes, definitions, and examples (see Appendix C). All interview transcripts and relevant quotes were carefully read again by one of the researchers (YS) to calculate the number of patients and percentage of total participants that had mentioned a specific theme at least once.

### Results

In total, 28 individuals (46.4% female, age: *M* = 53.97) participated in the interviews. The sample was ethnically diverse, with almost half of the participants coming from a minority background (*n* = 13, 46.4%): Non-Hispanic White: 53.6%, African American: 7.1%, Asian: 17.9%, Hispanic: 17.9%, other: 3.6%.

#### Characteristics of Lay Caregivers

**FIGURE 1:**
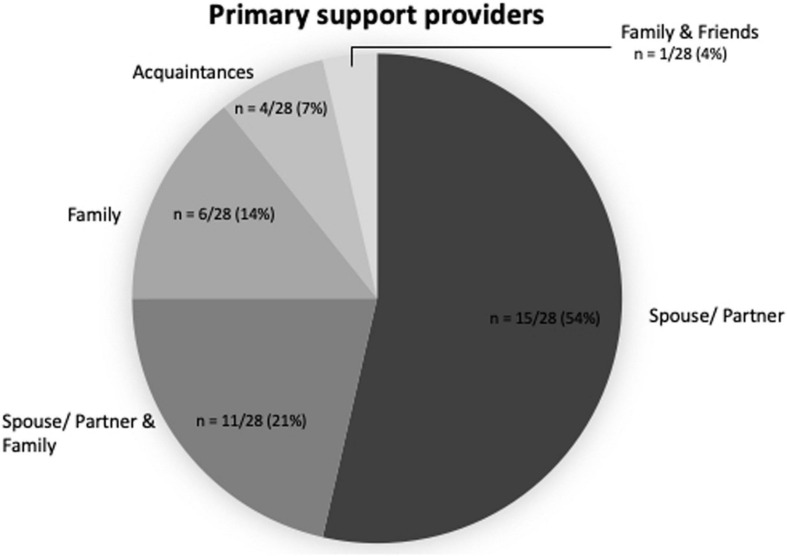
Primary support providers individuals after transplant (*n* = 28).

Lay caregivers, such as family members and friends, were the primary source of social support for transplant recipients. All participants reported receiving support from family members, friends, or acquaintances (*n* = 28/28, 100.0%). Moreover, about half of the participants reported to have at least one primary source of support, usually a spouse or partner, who was their main caregiver over the course of the transplant (*n* = 15/28, 53.6%, see Figure 1). For example, a participant mentioned that his wife supported him in taking his medications (P13, male). Another participant mentioned, “My husband does everything! He’s actually on top of everything more than I am.” (P07, female)

A considerable number of transplant recipients (*n* = 11/28, 39.3%) reported relying on several lay caregivers, such as the partner and other family members (*n* = 6/28, 21.4%), family members other than the partner (*n* = 4/28, 14.3%), or family members and friends (*n* = 1/28, 3.6%). One of the participants mentioned that his whole family ensures that he takes his medications regularly on time (P14, male). Another participant mentioned,

Well, yes my mother, I talk to her every day, three times a day, again my son, my boyfriend, (…) so yes, I have a lot of people supporting me, asking me this, did I do this. (P31, female)

Additionally, in two cases, acquaintances or non-family members supported individuals after transplant as lay caregivers (*n* = 2/28, 7.1%). One of them mentioned,

The helpers are mainly people from my [place of worship]. I have a woman who accompanies me to all my visits. Since my first transplant she has gone to every appointment with me. She brings lunch and meals and snacks. And uh, there’s a team of persons who provide me what I need in terms of food and stuff like that. So they’ve been very helpful. Um…I have a staff. A secretary, a bookkeeper, a staff in the kitchen. They do parts just to make sure. To get my car moved, get my mail, someone to go to the bank. So they take care [of] all those things. (P19, male)

Regardless of the type of their connection to the patient, patients described lay caregivers as essential for post-transplant recovery. For some patients after transplant (*n* = 4/28, 14.3%) this extended into experiencing new meaning in life, with life after transplant perceived as an opportunity to love and be with loved ones. A participant mentioned,

I have grown to love everything (…) I have taken advantage of time spent with my children, putting more attention to everything in life and being appreciative of God each and every day. (P23, female)

#### Types of Support From Lay Caregivers

Lay caregivers supported individuals after transplant in three main ways, instrumental, emotional, and informational support, with some patients noting some ambivalence about receiving support (see Table 3 for an overview). We will present each type of support with examples. Regarding *instrumental support*, lay caregivers had a wide range of tasks to help the patients with from daily living, financial matters to medication intake, to reduce the risk of infection in the immunosuppressed patients.

**TABLE 3:** An overview of the type of support, definition and number of patients reporting it (*N* = 28).

Types of support	Definition	*N* = 28 *n*, %
**Lay caregivers**		
Instrumental support:	Tangibly helping patients through taking relevant actions:	
For daily living	Providing support relevant to maintain daily lives of patients, including driving, cooking, getting groceries, and fulfilling daily practical needs	16, 57.1%
For financial matters	Supporting patients with expenses related to alloHCT treatment	4, 14.3%
For medication intake	Supporting patients with medication-related tasks, including taking medications, reminding of doses, refilling, and picking up the medications	26, 92.9%
Emotional support	Supporting patients by expressing words of encouragement, empathy and caring	11, 39.3%
Informational support	Lay caregivers were not the primary sources of informational support, but they helped as memory facilitators and conveyers of information from the healthcare providers	1, 3.6%
Ambivalence about receiving support	Support attempts that were not perceived as helpful or relevant to patients	7, 25%
**Healthcare Providers**		
Informational support:	Providing relevant information about survival and self-care after discharge	
Medications	Any relevant information about prescribed medications, including their functions, dosing information, side effects, and how to take them	23, 88.5%
Self-care	Information relevant for self-care (other than medication intake) included guidelines for nutrition and hydration	6, 23.1%
Support for medical needs	Helping patients practically to fulfill their medical needs through relevant actions (e.g., refill medications on time), which often made patients feel emotionally supported and cared for	18, 69.2%
Emotional support	Providing words of encouragement, making patients feel cared, which contributed to a trusting relationship between healthcare providers and patients	9, 34.6%

[My] husband does all the homework. He cooks, I don’t. Doctor doesn’t want me to have outside food. He reminds me to take the medication, he reminds me almost every time. (P11, female)

Instrumental support for daily living is vital as individuals after transplant must reduce their exposure to all possible sources of infection. This includes eating cooked foods only, watching what they drink and touch, and maintaining physical distance from people while managing side effects and other physical complications (Beattie and Lebel, 2011). Instrumental support for daily living from lay caregivers is crucial, including driving, cooking, getting groceries, and fulfilling daily practical needs (*n* = 16/28, 57.1%).

One participant mentioned,

Fortunately, my father helps me out with the transportation most of the time, so I don’t have to rely on public transportation. So, I would be infection free or decrease the chances of infection. (P20, male)

Another participant said that his wife makes sure to provide food and drinks that are nutritious and hydrating, yet very enjoyable and creative.

My wife was coming up with creative things, too. I started running out of ideas. She found things at Whole Foods like chicken potpie and roasted vegetable pot pie. I had a lot of stuff like that. It was very flavorful. (P10, male)

His wife also provided him with different drinks to make sure he stayed hydrated. “I’m very aware of having to hydrate because of these drugs. It’s also part because of my wife. She buys creative food things for recipes and different drinks.” (P10, male)

One participant suffered from stomach issues that made her nauseous, and her husband cooked food that was easy to swallow and digest. She said,

Husband does all the homework. He cooks, I don’t. Doctor doesn’t want me to have outside food. We eat together, every 2 to 3 hours I have to eat, unless I have a stomach problem. It’s the time when I am going to eat. Some snack: rice cake, I am [Asian]. (…) Every 2 hours I ate rice cooked with lots of water. Don’t have to chew; I was able to eat just a little bit, every 2 hours. (P11, female)

Instrumental support with financial matters is critical for patients as hematopoietic cell transplant is expensive and requires an extensive treatment process over a long period of time, often creating financial hardship for individuals and their families after transplant (Khera et al., 2014; Kim et al., 2015). Instrumental support with financial matters helped individuals to ameliorate their financial burdens, as well as their emotional stress and anxiety, as explicitly mentioned by some (*n* = 4/28, 14.3%). One mentioned, “I don’t even know where the bills are. I think he doesn’t want me to know because he doesn’t want me to worry.” (P07, female). Moreover, lay caregivers needed to balance the need for financial support with caring for individuals after transplant.

She has her own business, but she works from home. Financially we weren’t fantastic but no, she didn’t work a lot. She was kind of holding it all together. While I was at the hospital, she was not focusing on work at all. (P10, male)

Instrumental support for medication intake is another critical area in which patients need help, as individuals after transplant must take numerous medications even after a successful transplant and discharge from the hospital. Medication-related tasks are critical yet difficult for individuals after alloHCT due to the number and complexity of medications. Nearly all individuals in the sample (*n* = 26/28, 92.9%) mentioned that their lay caregivers helped with medication-related tasks, such as taking medications, reminding of doses, refilling, and picking up the medications.

Every 2 and 3 hours I have to take the medication. I have to be aware of the time and take and my husband reminds me. In 6 months, I have only missed one tablet. (P11, female)

She also mentioned that she was able to refill medications on time because of her partner’s support: “[My] husband calls the pharmacy, they send us FedEx.” (P11, female)

***Emotional support***, such as expressing encouragement, empathy and caring, plays a significant role in helping individuals after transplant as patients after alloHCT become physically and emotionally vulnerable (Rini et al., 2011). More than half of the participants reported receiving emotional support (*n* = 11/28, 39.3%). “As soon as I went into the hospital, my husband put up a website for me because everyone called. So many people wrote in, it was wonderful” (P07, female). Internet and technology development have also facilitated emotional support. “I stay home, but I’m not alone a lot. I do have friends who drop in a lot. We have a lot of support. And I Skype a lot, everybody has that all over the world.” (P07, female)

For emotional support, individuals after transplant rely on a broader range of support sources than for instrumental support, including members of their extended family, friends, and acquaintances. In one case, an individual relied more on friends for emotional support than family members and distinguished the different types of support she received.

They really support me in every way, my friends and my family. But for the medication, it’s my family, my husband and my little ones, they remind me all the time, but emotionally, my friends, they help me, like when I’m not feeling too up to it, or if I don’t have, like, enough energy they help me a lot. (P22, female)

Individuals after transplant also perceive the importance of receiving emotional support. An individual after transplant suggested to other patients,

To be able to talk about your problems, talk with friends and family. I would [be] trying to get them involved in a support group. I am lucky to have a very supportive family and friends, so I didn’t have to go out of my shell. You want people to ask how you are doing. You need someone who is patient enough to listen. (P07, female)

*Informational support* was not reported from **lay caregivers (*n* = 0.0%) as** healthcare providers were the primary source of informational support, but lay caregivers helped as memory facilitators and conveyers of information from the healthcare providers. In one interview the caregiver interrupted to provide the correct response when the patient was asked what medications he had to take on an empty stomach. The wife interrupted that “the only one that he takes on an empty stomach is Prilosec” (P25, male). This response indicates that she helped the patient to remember the information. One patient (*n* = 1/28, 3.6%) mentioned her “husband keeps asking doctors about [side effects of medications]” (P07, female). In this case, the patient’s husband helped the patient by clarifying and reiterating the information received from the doctors and also asked further questions that the patient might have missed or forgotten to ask. All other participants sought information either by using online resources or directly asking their healthcare providers. One person mentioned, “most of the teaching [is through] the doctors, and the nurses. And I also go online sometimes and do my little own research.” (P22, female)

Support attempts were not helpful to all individuals and some patients expressed *ambivalence about support* from others (*n* = 7/28, 25%). One participant chose not to share his situation with the people around him at all, believing that it would only cause more trouble.

I’m a public figure, I kept it secret for a long time, (…) Keeping it secret: if I told them they were going to worry. They would want me to take medication they know about. Take this, take that. I [had] not wanted to deal with all that advice. Listen, I have my doctors. You guys, just pray, don’t try to be my doctors. (P19, male)

Four patients expressed that they did not like the feeling of being monitored by their lay caregivers. They sought independence and control over their situation, though to no avail. In these cases, less support may be better. They perceived actions of support not as helpful, but somewhat unpleasant and even troublesome. An individual mentioned,

My wife asks me all the time whether I’m taking medications, whether I took the medications, but basically I just think she’s asking too much because I’m just taking it. (P13, male)

One mentioned that he does not take medications when he is bothered by other people (P17, male). The degree of support received, and the quality of relationships varied among individuals.

In another case, the support from lay caregivers was ambiguously helpful. A participant mentioned the clothes she is accustomed to wearing:

My sister doesn’t want me to use that, she buys new clothes. I want the old clothes. She said I cannot use it anymore. Where is it what I like. Even [the clothes] is old [in] our thinking, I will go to the dry cleaning, wash and dry clean. I don’t know where the clothes are. She is hiding clothes away. I don’t know where they are (P29, female).

#### Characteristics of Healthcare Providers

We found that all participants asked about healthcare providers identified them as a source of support (100% of 26 interviews). Participants referred specifically to doctors and nurses when speaking about healthcare providers. At the hospital, clinical interdisciplinary teams worked closely together. Therefore, most individuals after transplant perceived them as a team (*n* = 22/26, 84.6%).

Nearly all individuals reported having a good relationship with their healthcare providers and evaluated the relationship as positive (*n* = 24/26, 92.3%). The quality of the patient-healthcare provider relationship influenced individuals’ health behaviors, especially with regards to making medical decisions. When patients built trusting relationships with their healthcare providers, they felt confident following their healthcare providers’ guidelines. One individual mentioned, “I’m a believer, I believe in him. First thing we do in any situation that you want to get help you have to believe. I believe in him. So what he says is right.” (P17, male) Another individual also mentioned,

Very simply, they have my best intentions in mind, and they override what do you call it. they know what the right thing to do is, whether I like it or not. So they’re not sticking me with bad tasting medications on purpose to make me feel bad. This is the right thing to take. They’re the experts, they know the conditions, they know what’s coming. (P13, male)

At the same time, one participant (*n* = 1/26, 3.8%), while mentioning he did receive some support from his healthcare providers, also expressed that he had received too little information at the discharge meeting and was therefore dissatisfied with the healthcare providers. He said,

I ended up back in here [admitted to the hospital]. They didn’t talk about hydration. (…) Hydration is likely a bigger deal than they tell you about. I was back in for 12 days. I was berserk about that. (P01, male)

Another patient was ambiguous in her response regarding the relationship with healthcare providers and did not quite perceive a relationship: “Well, I have not had problems with them. I don’t care.” (P12, female)

#### Types of Support From Healthcare Providers

Individuals after transplant perceived that their healthcare providers mostly provided informational support in line with their expertise. However, many individuals mentioned that they also received other types of support from the healthcare providers, including emotional support. We will present specific examples detailing the kinds of support provided and how they helped patients after transplant (see Table 3 for an overview).

Individuals after transplant mostly received *informational support* from healthcare providers (*n* = 23/26, 88.5%). A wide spectrum of healthcare providers, including pharmacists, dietitians, hematologists and specialist nurses, provided informational support verbally and in writing. Patients received information on medications (*n* = 23/26, 88.5%) and nutrition, including hydration (*n* = 6/26, 23.1%).

One participant reported that the relationship with healthcare providers made it easier to take medications.

Because they tell me exactly that I need to take them, why I need to take them, yeah always teaching. Always teaching, yup. Every single day, every single appointment. The nurse coming first goes over all of the medication and the doctor will do the same thing. (P22, female)

Many patients reported that their health providers met their *medical needs* (*n* = 18/26, 69.2%). One individual mentioned, “They make sure that all my medical needs are met so I can recover and go back to a regular life.” (P14, male). Another patient recalled the help she received when she forgot to refill her medications on time and needed an immediate supply. “I have run out of the Prograf and that is very important, but the doctors called in a 4-day refill script to [name of pharmacy 6] and I picked it up.” (P31, female). Often, support for medical needs was combined with emotional support, as a female participant reported in dealing with her difficult stage four GvHD. She said,

One of the reasons, I have to tell you, are the [name of hospital 1] nurses. They are angels. They packed me in ice at night. They looked at me, my skin was peeling, you couldn’t touch me any place without me screaming because it hurt so much. It’s very good to have nurses that are so kind. They actually stayed with me. They didn’t have to do more than just come when I rang the bell. It’s harder at night because everything is so quiet, and nothing can distract you from the pain. (P07, female)

Healthcare providers, usually nurses, were also the sources of *emotional support*, encouraging individuals after transplant and showing care for their personal lives (*n* = 9/26, 34.6%). The attitude of healthcare providers and the way individuals after transplant perceived them influenced them in their recovery process. As one participant noted,

In the sense that they know things are important, they make me feel like my recovery is important, they make sure that all my medical needs are met so I can recover and go back to a regular life. (P14, male)

Another participant said,

I think the best thing was speaking with all the nurses. Becoming friendly with them. (…) Yeah, and the day I left the hospital was my birthday and they brought me a birthday cake. I can’t believe they did that! So that was nice, that brightened up my day. (P43, female)

Emotional support contributed to a good and trusting relationship with healthcare providers, encouraging and helping individuals after transplant to recover. None of the patients expressed *ambivalence about support* from healthcare providers (*n* = 0/28, 0%).

### Discussion

Study 2 explored the sources and functions of social support for adherence to recommended self-care in individuals after alloHCT. In line with a previous study, the role of lay caregivers is critical for patients after alloHCT as they closely support patients with managing home care and other daily tasks after transplant (Posluszny et al., 2018). Moreover, healthcare providers played an important role in providing information regarding medications and self-care after discharge. They supported patients with medical needs and also cared and comforted them. Our findings suggest that these support sources serve complementary functions. While emotional and instrumental support is largely within the domain of lay caregivers, medical needs and informational support are mostly provided by healthcare providers. Thus, patients must coordinate support-seeking from both lay caregivers and healthcare providers to facilitate multiple medication intake and self-care after alloHCT.

## General Discussion

The current study examined the importance of social support in patients after alloHCT. Both quantitative and qualitative data suggest the importance of social support to enable patients to maintain a complex self-care regimen related to infection prophylaxis and survival. Study 1 found that the presence of a dedicated lay caregiver is related to improved chances of survival after alloHCT, supporting previous findings (Beattie et al., 2013; Foster et al., 2013; Ehrlich et al., 2016; Harashima et al., 2019) and Study 2 examined the different types of support provided by lay caregivers and healthcare professionals. Our findings suggest that lay caregivers and healthcare providers each serve the patients in different ways. Moreover, the different sources and networks of social support in Study 2 (see Figure 1) suggest that information on marital status misses the contribution of other lay caregivers, which may explain why these studies found no link between marital status and survival (Gerull et al., 2017; Sato et al., 2018; Tay et al., 2020). More nuanced measures of support than marital status should be considered for future research.

The difficulties of recovery after transplant, often characterized by severe complications, patients feeling unwell, fatigued, and socially limited due to their immunosuppressed state, make caregiver support essential for day-to-day living (So et al., 2003; Soubani, 2006; Rini et al., 2011; Wulff-Burchfield et al., 2013; Posluszny et al., 2018). Financial support seems essential for patients after alloHCT (Preussler et al., 2019). Our culturally diverse sample showed a uniformly high reliance on social support, although ethnicity and cultural background played a role in providing appropriate concrete supportive acts (e.g., support for adequate nutrition and hydration with buying bread and soft drinks vs. cooking rice with lots of water). Furthermore, caregivers’ presence motivated patients and gave them reasons to live despite their difficult health condition, supporting prior research (Krause, 2007). We assume that this mechanism is also true for lay caregivers who were shouldering a high caregiver burden in meeting the considerable physical, financial, and emotional needs of individuals after transplant. The presence of their loved ones, despite their worsened health condition, may give meaning to caregivers’ own lives. Efforts made by healthcare providers to support patients were deeply appreciated by participants. In line with prior research, caring and trusting patient-provider relationships foster patients’ adherence to recommended self-care (Haskard Zolnierek and DiMatteo, 2009; Hillen et al., 2010; Espinosa and Kadić-Maglajlić, 2019).

There are several limitations to these studies. First, both studies were conducted in a single treatment center in a large urban area over a limited period of time. The findings may not be generalizable beyond this setting and time period. However, the diversity of the sample regarding ethnic background and age range makes it likely that the findings will be more widely applicable. Second, Study 2 may show some sampling biases, as participants who volunteered to enroll and be interviewed could differ from those not enrolled or interviewed (e.g., in health or adherence). We tried to limit bias by inviting everyone who was eligible and scheduled to receive an alloHCT. Another potential bias is that individuals may have provided socially desirable responses during the interviews. However, participants were highly motivated and passionate about contributing to the study to improve care for future transplant recipients and often contributed more than we asked (e.g., by sending pictures of their numerous pill containers). Therefore, we assume that many participants responded with earnestness and sincerity. In future research, interviewing the caregiver could give an additional validation of patients’ reports and a complementary perspective (Posluszny et al., 2018). Third, the treatment center we collaborated with required nominating a dedicated support provider in order to receive an alloHCT, as is common practice (National Marrow Donor Program, 2017; Preussler et al., 2019). Thus, the majority of patients in Study 1 and 2 had a dedicated caregiver, and only a small group of patients had limited or no support. Future studies in treatment centers which do not require a caregiver may observe stronger effect sizes than those observed in this study. However, we observed variability in support in Study 1 and 2; despite the efforts of the hospital, some caregivers do not fulfill their role. Anecdotally, during data collection for Study 2, we received consent from two individuals whose lay caregiver support was minimal, and both patients passed away before we could interview them. These two cases, in addition to our observations of the high support needs of our participants, illustrate the difficulty of adhering to the self-care recommendations and the multiple medication regimen without a rigorous support system, with severe consequences for survival. Fourth, in the chart review in Study 1 the research team was able to merely establish the presence of a caregiver but was not able to determine more information about the quality of support (e.g., if there were other caregivers involved and with what frequency and duration caregivers were available, as others have done; Foster et al., 2013). In Study 2, we were able to describe the sources and functions of support in detail, but the sample was too small to examine the link with survival. Therefore, the conceptual linkages between types of support and impact on survival are merely suggestive and require further research.

Despite these limitations, this article has several strengths. First, this article contributes to previous literature that support truly matters for survival (Beattie et al., 2013; Foster et al., 2013), using both quantitative and qualitative data. Study 1 underscored the essential role of support, while Study 2 provided a nuanced description of the characteristics of support providers and types of support. The availability of support may influence survival in two ways. Support may impact adherence to a complex self-care regimen with quality of life. Support may also help patients find meaning in life and carry on despite facing a high risk of mortality and challenges with quality of life. Second, this article contributes to the evidence base of a less frequently studied cancer population (i.e., blood and lymphoid cancer patients after alloHCT), where there is to our knowledge just one other study of patient-caregiver responsibilities (Posluszny et al., 2018). Finally, to facilitate adherence to this complex self-care regimen, all three parties—the transplant recipient, lay caregivers, and the healthcare providers—must work together as a team. A prior systematic literature review showed that social support from family members increases medication adherence (DiMatteo, 2004), but does not focus on healthcare providers. Our findings suggest the importance of support from healthcare providers who should be considered part of the patient support team.

**FIGURE 2:**
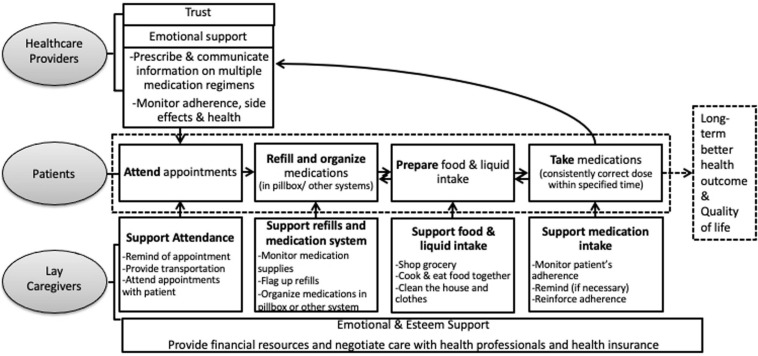
Cascade of social support tasks to ensure adequate self-care.

Based on our findings, we propose a process model of social support for patients who need to perform a complex self-care regimen, which needs to be tested in future studies and could inform intervention development (see Figure 2). The figure represents the interactions among the support triad of healthcare providers, lay caregivers, and patients, and the functions of social support from each source. Based on a strong foundation of trust, healthcare providers are mainly responsible for providing professional informational support regarding prescribed medications and medical needs, and effectively communicating this information to patients and lay caregivers. Lay caregivers provide more intimate, daily instrumental support, such as refilling and organizing medications, managing financial matters, cooking, and helping with physical distancing. They are also the major sources of emotional support, encouraging and comforting patients, which helped patients to maintain a positive and optimistic attitude toward their recovery. We propose that the different types of support are partly co-occurring and intertwined (e.g., with actions of practical support expressing a lay caregiver’s love for the patient) and that there may be an interactive effect between different types of support that deserves further research.

Our findings—although they are based on a limited sample size from a single transplant center—highlight the importance of measures that many transplant centers already implement in their clinical care to ensure adequate support for individuals after transplant. To ensure that patients and caregivers can follow the complex self-care regimen, healthcare providers need to devote time and attention to conveying the details about the relevant self-care tasks. Although transplant clinics already provide relevant education sessions to patients and caregivers, consistent implementation is critical, and it might be also helpful to repeatedly check on the availability of caregiver support throughout treatment even after discharge (Preussler et al., 2019). A scheduled dedicated prescription meeting delivered by a nurse or a pharmacist in the presence of both patient and caregiver was helpful to the participants in this study who received it. Second, the requirement to nominate a dedicated lay caregiver after transplant seems to be warranted. Third, social support is essential for following a self-care regimen. If providers of social support cannot attend the prescription meetings, they should be informed about the patient’s support needs via a phone call or at least by a written letter. Lastly, we suggest the need for more clinical interventions and possibly policy implementations as some question whether poor social support should keep patients from receiving a transplant (Sharma and Johnson, 2019).

## Conclusion

In conclusion, this study contributes to a more detailed theoretical and practical understanding of social support for a complex self-care regimen. To the best of our knowledge, this is amongst the first in-depth investigation of support, survival and self-care in patients after alloHCT. The teamwork of patients, lay caregivers, and healthcare providers is the basis for successful survival after transplant. Based on our findings, healthcare providers and lay caregivers must work in tandem to promote adherence to self-care regimens. The results of this study also suggest that there is a need to develop interventions for patients and their caregivers to facilitate treatment adherence for survival.

## Data Availability Statement

The datasets for these studies are available from the authors on request.

## Ethics Statement

The studies involving human participants were reviewed and approved by the local Institutional Review Board at Mount Sinai School of Medicine reviewed and approved both studies (Study 1: HS# 13-00761 and Study 2: HS# 12-00453). The participants provided their written informed consent to participate in this study.

## Author Contributions

GS designed the research project. ES and WHR advised with designing the study and also helped conduct the study. GS and YS developed the research questions. GS analyzed the data for Study 1. YS and SC helped with data collection and analyzed the data for Study 2. GS wrote Study 1 and YS wrote Study 2. YS, SC, JR, and GS contributed to writing and revising the manuscript. YS, JR, and GS revised the manuscript for the final submission. All authors contributed and provided revisions of the manuscript.

## Conflict of Interest

The authors declare that the research was conducted in the absence of any commercial or financial relationships that could be construed as a potential conflict of interest.
