# Supplementary material for: It Takes a Team to Make It Through: The Role of Social Support for Survival and Self-Care After Allogeneic Hematopoietic Stem Cell Transplant
Source: Front Psychol. 2021 Mar 26;12:624906. doi: 10.3389/fpsyg.2021.624906 (PMC8044751; doi:10.3389/fpsyg.2021.624906)
Supplement: Supplementary file 1 [file Data_Sheet_1.docx]

Appendix A

Consolidated Criteria for Reporting Qualitative Research

(COREQ) Checklist

1. Interviewer/ facilitator: Which author/s conducted the interview or focus group?

The authors, including YS, SC and the principal investigator (GS), and six other research assistants in the team conducted the interviews.

2. Credentials: What were the researcher's credentials? E.g. PhD, MD

The principal investigator holds a Ph.D. in psychology. Two authors (YS and SC) and six other research assistants were all female with either a bachelor’s or master’s degree in social sciences, public health or health sciences at the time of the study.

3. Occupation: What was their occupation at the time of the study?

The principal investigator was working as a research scientist at Columbia University, and the other members in the research team were either graduate students or recent graduates preparing for graduate schools.

4. Gender: Was the researcher male or female?

The interviewers were all female.

5. Experience and training: What experience or training did the researcher have?

The principal investigator is an expert in health psychology and has conducted several studies of frequent health behaviors with people living with and without chronic conditions. Interviewers were trained by the principal investigator with the interview guide and using role-play within the research team. Then interviewers shadowed interviews prior to conducting interviews with participants in the study.

6. Relationship established: Was a relationship established prior to study commencement?

There was no relationship between interviewers and participants prior to the study. Rather, relationships were built over the course of the study, beginning with recruitment and consenting by clinical staff, continuing with handing out the pillbox before or shortly after transplant in a research meeting before discharge home, and weekly contact after hospital discharge between research team and participants. By the time the interviews were conducted at 3 and 6 months after the first hospital discharge, participants had gotten to know the research team members well. Some participants sent us pictures of their table full of medications and also contacted the research team on their own via email and study phone with questions and for study logistics.

7. Participant knowledge of the interviewer: What did the participants know about the researchers?

Participants knew that all research team members were staff or students at Columbia University or Mount Sinai. Participants were aware that the research team included Spanish and also Mandarin speakers. Participants were also made aware that everything they shared with research team members would be kept confidential and would not affect their treatment.

8. Interviewer characteristics: What characteristics were reported about the interviewer/ facilitator?

The interviewers were an ethnically diverse group, with a nuanced understanding of different cultures. They were also fully aware of and trained in the ethics of conducting research, including maintaining strict confidentiality. Throughout the study, the interviewers tried not to be biased or look for specific results. Usually two interviewers conducted the interviews, with one interviewer mainly conducting the interview and the other audio recording and taking notes and helping to add probing questions and to keep the flow of the interview.

9. Methodological orientation and theory: What methodological orientation was stated to underpin the study? (e.g. grounded theory, discourse analysis, ethnography, phenomenology, content analysis)

Thematic analysis (Braun & Clarke, 2006)

10. Sampling: How were participants selected?

We used nonprobability sampling and participants were recruited from all patients undergoing alloHCT at a single urban treatment center from 08/15/2012 to 07/15/2014.

11. Method of approach: How were participants approached?

Clinical staff members informed patients scheduled for alloHCT of the study, either during pre-transplant outpatient visits prior to admission for transplant or during their hospitalization after transplant and obtained informed consent from interested eligible patients.

12. Sample size: How many participants were in the study?

In total, 28 individuals (46.4% female, age: *M* = 53.97) participated. The sample was ethnically diverse, with almost half of the participants coming from a minority background (*n* = 13, 46.4%), (Non-Hispanic White: 53.6%, African American: 7.1%, Asian: 17.9%, Hispanic: 17.9%, other: 3.6%). Two individuals answered some of the interview questions but not all: Patients 20 and 21 could not complete the section on healthcare providers due to time constraints and health conditions. Most of the interviews were conducted in English (n = 26, 92.9%), some in Spanish (n = 2, 7.1%), and none in Mandarin because some Chinese-American participants preferred English as their interview language.

13. Non-participation: How many people refused to participate or dropped out? Reasons?

Interviews from five patients were missing: Three patients were deceased, one patient was unavailable, and the other was too ill to participate in the interview.

14. Setting of data collection: Where was the data collected?

The study was conducted at a urban hospital. This hospital’s department of hematology and medical oncology has a long history of treating hematological malignancies and cancer. The location of the study site allowed diverse individuals to receive treatment. Interviews were conducted face-to-face in the hospital while participants were waiting for their clinical appointments or through phone interview depending on participant preferences.

15. Presence of non-participants: Was anyone else present besides the participants and researchers?

In some interviews, the caregivers were present with the participant as they accompanied the participants for hospital appointments.

16. Description of sample: What are the important characteristics of the sample?

Eligible participants were adults with leukemia or lymphoma cancers who had received an alloHCT at the hospital. Participants were eligible for the study if they were 18 years or older and spoke and read English, Spanish or Mandarin.

17. Interview guide: Were questions, prompts, guides provided by the authors? Was it pilot tested?

A theory-driven interview guide (see Appendix A) was developed by the principal investigator, who is an expert in health psychology and has conducted many studies in this area. The interview covered various topics centered around adherence and home care, including questions on general medication adherence, knowledge of medications, skills, social motivation and adherence strategies, including use of an electronic pillbox. Interviewers were trained by the principal investigator; the interview questions were pilot tested within the research team meetings and also during role-play interviews prior to conducting actual interviews with patients, and interviewers were also allowed to shadow interviews before the interviewers conducted interviews themselves.

18. Repeat interviews: Were repeat interviews carried out? If yes, how many?

The research team conducted repeat interviews with 13 participants at 3 and 6 months after first hospital discharge, and single interviews for 15 participants, with 10 of these being interviewed at 3 months and 5 at 6 months after hospital discharge.

19. Audio/ visual recording: Did the research use audio or visual recording to collect the data?

All interviews were audio recorded.

20. Field notes: Were field notes made during and/or after the interview or focus group?

The research team took field notes for each contact with participants including during the interviews.

21. Duration: What was the duration of the interviews or focus group?

The interview lasted about 40 to 60 minutes.

22. Data saturation: Was data saturation discussed?

The research team discussed data saturation and decided to continue recruitment up to 30 patients to obtain enough data to reflect the multifaceted experiences in this diverse patient population.

23. Transcripts returned: Were transcripts returned to participants for comment and /or correction?

No, interviews were transcribed and given to the interviewers who had conducted the interviews for comment and correction, but not to participants.

24. Number of data coders: How many data coders coded the data?

Two individuals coded the data (YS and SC).

25. Description of the coding tree: Did authors provide a description of the coding tree?

A codebook, based on the relevant theoretical frameworks, including the Information-Motivation-Behavioral Skills model and classical social support theory, was developed under the supervision of the principal investigator. The researchers used both theory-driven and data-driven coding. For paper 1, it was mostly data-driven coding as the researchers extracted relevant quotes on pillbox use, then later identified and categorized the quotes in different themes. For paper 2 and 3 researchers started out with theory-driven coding, but we also applied data-driven coding along the way and identified additional emerging themes. Codes were modified and added as the researchers were reading through the transcripts. Over informed discussions between research assistants and the principal investigator, we refined the codebook with themes, definitions and examples (see Appendix B).

26. Derivation of themes: Were themes identified in advance of derivation from the data?

Yes, themes were identified based on the theoretical framework, but researchers also found some additional emerging themes from the interview that were not identified in the original theoretical model.

27. Software: what software, if applicable, was used to manage the data?

The coders (YS and SC) initially used Excel spreadsheet, and transferred all data into NVivo version 11 later to store, search and code the interviews in a more systematic way.

28. Participant checking reporting: did participants provide feedback on the findings?

No.

29. Quotations presented: Were participant quotations presented to illustrate the themes/findings? Was each quotation identified?

Yes, participant quotations were included throughout the manuscript with participant number, age and gender.

30. Data and findings consistent: Was there consistency between the data presented and the findings?

Several authors checked that the findings presented reflected the data with the intent to avoid drawing any unwarranted conclusions that are not supported by the data. Intercoder reliability scores are provided throughout the manuscript.

31. Clarity of major themes: Were major themes clearly presented in the findings?

The findings in the manuscript are reported based on the major themes from the data.

32. Clarity of minor themes: Is there a description of diverse cases or discussion of minor themes?

No.
