# Supplementary material for: It Takes a Team to Make It Through: The Role of Social Support for Survival and Self-Care After Allogeneic Hematopoietic Stem Cell Transplant
Source: Front Psychol. 2021 Mar 26;12:624906. doi: 10.3389/fpsyg.2021.624906 (PMC8044751; doi:10.3389/fpsyg.2021.624906)
Supplement: Supplementary file 2 [file Data_Sheet_2.docx]

Appendix B

# Qualitative Interview Guide

Multi-Method Study of Cancer Patients' Medication Adherence
After Allogeneic HSCT

Formative Research - In-Depth Interviews

**Background Information**

Please tell me a little bit about yourself.

*Probes:*

a. Where are you from? Where do you live? How long have you lived in [New York City]?

b. How long have you been living with cancer?

Can you share with me a little bit about how life has changed for you since your transplant?

*Probes:*

a. What is the best thing and worst thing that has happened since transplant?

b. Has the transplant impacted your daily routines and/or behaviors? How so?

c. Do you ever feel like you have a hard time managing your cancer and the transplant treatment? Can you tell me more about that?

**Social Motivation and Support**

Who in your life knows that you are taking medication? (Probe for partners and other family members, friends known in person and online/Facebook friends, religious community, coworkers, roommates, etc.)

Probes: a. Are there people in your life who support you taking your medications? Are there people who do not support you taking your medication?

b. Are there people in your life who help to make sure that you take your medication regularly/on time?

Are there people in your life who make it difficult for you to make sure that you take your medication regularly/on time?

How have your eating and drinking habits changed since the transplant?

*Probe:*

a. What changes have you made in order to facilitate eating and drinking during the day?

b. What do you eat on a typical day – for breakfast? For lunch? For dinner? As snack? How often and how much do you eat?

c. What do you drink on a typical day? Do you like some beverages better than others (for example tap water vs carbonated drinks?) How often and how much do you drink? Do you drink alcohol? How often and how much? What types of alcohol?

d. Does feeling ill, such as nauseated, ever make it difficult to eat and/or drink during the day? How so? What have you done about it?

Does a lack of money (i.e., end of a paycheck, periods of unemployment, a delayed government assistance check, lack of help from friends and family, etc.) ever make it difficult for you to take your medication? Can you tell me about this? What do you do about it?

Does anyone help you with the pillbox? Who, specifically, helps you use the pillbox? How do they help you?

*Probe:*

Do they help you refill the box? Do they help you get medications out of the box?

Does anyone make it difficult for you to use the box? How so? What do you do about this?

**Attitudes toward Healthcare Providers**

*Now I would like to ask you some question about your experiences receiving health care.*

Tell me about your health care provider(s)?

Probes: a. Who is the person who primarily treats you?

b. What is your relationship like with your care provider? Do you think your relationship with your care provider makes it easier or harder to take your medication? How so?

c. Do you feel like your care provider understands your needs? [for minority patients: What about as a Black, Latino/Latina, Asian man/woman?]?

*Thank you for your participation and for sharing your experiences with me today. We are at the conclusion of the interview, is there anything else related to your experiences that you would like to add?*
