# Supplementary material for: It Takes a Team to Make It Through: The Role of Social Support for Survival and Self-Care After Allogeneic Hematopoietic Stem Cell Transplant
Source: Front Psychol. 2021 Mar 26;12:624906. doi: 10.3389/fpsyg.2021.624906 (PMC8044751; doi:10.3389/fpsyg.2021.624906)
Supplement: Supplementary file 3 [file Data_Sheet_3.docx]

## Appendix C

# Interview Coding Guide

| **Code** | **Definition** | **General guidelines and potential questions that target the code (but responses can be in elsewhere in the transcript)** | **Example quotes** |
| --- | --- | --- | --- |
| Meaning in life | Positive attitude that helps one to make meaning about their life after transplant and situations | Can you share with us how your life has changed after the transplant? | “I have grown to love everything. (…) For example, living day by day, I have taken advantage of time spent with my children, putting more attention to everything in life and being appreciative of god each and every day.” (P23, female).  “I needed to get well. The family understood, they knew what was happening. You take it out on your husband and your children.” (P7, female). |
| Lay caregivers- instrumental support:  -Daily life related,  -Medication related | Family members or friends provide tangible ("doing something physical/ beyond emotional level" (Cutrona)) support related to daily tasks (such as cooking, completing house chores, shopping for groceries, and transportation), and medication related | Who in your life knows that you are taking medication? Are there people in your life who support you taking your medications? Are there people who do not support you taking your medication? | “My partner did the cooking, he works from home, he did all of that.” (P16, male)  “My wife does bulk of it (reminding to take medications). (…) She’ll ask me if I have and she’s the one that makes sure I’m taking my medicine.” (P26, male). |
| Lay caregivers- financial matters | Any mentioning on financial struggles patients experienced. Family members or friends provide financial support. | Does a lack of money (i.e., end of a paycheck, periods of unemployment, a delayed government assistance check, lack of help from friends and family, etc.) ever make it difficult for you to take your medication? Can you tell me about this? What do you do about it? | “I don’t even know where the bills are. I think he doesn’t want me to know because he doesn’t want me to worry.” (P07, female) |
| Lay caregivers- emotional support | Family members of friends provide words of encouragement through verbal conversations or written communication, providing comfort, caring | Are there people in your life who support you taking your medications? Are there people who do not support you taking your medication? | “My husband put up a website for me because everyone called. So many people wrote in, it was wonderful.” (P7, female). |
| Lay caregivers-informational support | Family members of friends provide information relevant to treatment, medical procedures and medications | Are there people in your life who support you taking your medications? Are there people who do not support you taking your medication? |  |
| Healthcare provider-support with medical needs | Health care providers attending to patients with medical needs | Tell me about your health care provider(s)? Do you feel like your care provider understands your needs? | “They make sure that all my medical needs are met so I can recover and go back to a regular life.” (P14, male)  “He is a very good professional. (…) My doctor understood my needs and the communication was clear and important.” (P23, female) |
| Healthcare provider- emotional needs | Support focusing on emotions (i.e. words of encouragement, staying together, providing comfort) from health care providers | Tell me about your health care provider(s)? Probes: a. Who is the person who primarily treats you? b. What is your relationship like with your care provider? Do you think your relationship with your care provider makes it easier or harder to take your medication? How so? c. Do you feel like your care provider understands your needs? | “I think the best thing was speaking with all the nurses. Becoming friendly with them. (…) Yeah, and the day I left the hospital was my birthday and they brought me a birthday cake. I can’t believe they did that! So that was nice, that brightened up my day.” (P43, female) |
| Healthcare provider- informational support:  -medication related,  -diet and hydration | Support focusing on providing information regarding medications, and nutrition/ hydration. | Tell me about your health care provider(s)? Probes: a. Who is the person who primarily treats you? b. What is your relationship like with your care provider? Do you think your relationship with your care provider makes it easier or harder to take your medication? How so?  How have your eating and drinking habits changed since the transplant? What do you eat/ drink on a typical day – for breakfast? For lunch? For dinner? As snack? How often and how much do you eat? | “Because they tell me exactly that I need to take them, why I need to take them, yeah always teaching. Always teaching, yup. Every single day, every single appointment. The nurse coming first goes over all of the medication and the doctor will do the same thing.” (P22, female)  “I followed low microbial diet, no immunogenic food (…) I followed what they recommended, 95% of meals were home cooked.” (P16, male) |
| Ambivalence about support | Patient feeling confused or ambiguous about the received support (lack of or insufficient support) from lay caregivers or healthcare providers | Are there people in your life who make it difficult for you to make sure that you take your medication regularly/on time?  Tell me about your health care provider(s)? Probes: a. Who is the person who primarily treats you? b. What is your relationship like with your care provider? Do you think your relationship with your care provider makes it easier or harder to take your medication? How so? c. Do you feel like your care provider understands your needs? | “It depends, they watch me. I’m under observation.” (P17, male). “Keeping it secret: if I told them they were going to worry. They would want me to take medication they know about. Take this, take that. I not wanted to deal with all that advice. Listen, I have my doctors. You guys, just pray, don’t try to be my doctors. This remedy comes from the family. They send me home remedies. Well, they didn’t listen. Their kindness shows.” (P19, male) |
